# Novel 5, 6-Dihydropyrrolo[2,1-a]isoquinolines as Scaffolds for Synthesis of Lamellarin Analogues

**DOI:** 10.1155/2011/103425

**Published:** 2011-08-07

**Authors:** Shao Han Liao, Dai Hua Hu, Ai Ling Wang, De Peng Li

**Affiliations:** ^1^College of Environment and Chemical Engineering, Dalian University, Dalian 116622, China; ^2^Liaoning Key Laboratory of Bio-Organic Chemistry, Dalian University, Dalian 116622, China; ^3^College of Agronomy, Northwest A&F University, Yangling 712100, China

## Abstract

As core skeletons of lamellarins: 5,6-Dihydropyrrolo[2,1-a]isoquinolines are one of the important alkaloids that exhibit significant biological activities, in this study, an efficient synthetic route was described for two novel compounds, 5,6-dihydropyrrolo[2,1-a]isoquinolines **I** and **II**. Compound **I** was synthesized from isovanillin with 28.3% overall yield by a six-step reaction while **II** from 2-(3, 4-dimethoxyphenyl) ethanamine was with 61.6% overall yield by a three-step reaction. And the structures of these two compounds were confirmed by means of IR spectrum, ^1^H NMR, ^13^C NMR, MS, HRMS, and melting point measurements.

## 1. Introduction

Lamellarins are a group of hexacyclic marine alkaloids that were initially isolated from a prosobranch mollusk by Faulkner and coworkers in 1985 [1]. Since then, over 70 compounds belonging to this group have been isolated and identified [2].

Some of these lamellarins and related compounds exhibit interesting biological activities in multidrug resistance (MDR) and their corresponding parental cell lines [3]. As well known [4], Lamellarin D (LMD) exhibits a significant cytotoxicity against a large panel of cancer cell lines and is a potential non-CPT (camptothecin) topoisomerase 1 poison [5, 6]. LMD affects cell cycle and acts on cancer cell mitochondria to induce apoptosia [7].

Due to the fascinating novel structures and biological activities, more and more researchers have devoted into the synthetic studies of lamellarins [8] and related 3,4-diarylpyrrolo derivatives. As one of the important alkaloids, 5,6-Dihydropyrrolo[2,1-a]isoquinolines exhibits pronounced biological activities. The biological activity of 5,6-dihydropyrrolo[2,1-a]isoquinolines **I** and **II** was evaluated by their effects on the proliferation of MDA-MB-231 (breast cancer cell line) by MTT assay. Our results showed that compound **I** could significantly inhibit the proliferation of MDA-MB-231 at the concentration of 40 *μ*g/mL, in contrast, compound **II** could enhance the proliferation of the MDA-MB-231 at the same concentration. In addition, they are also scaffolds for synthesis of lamellarin analogues [9].

**Scheme 1:**
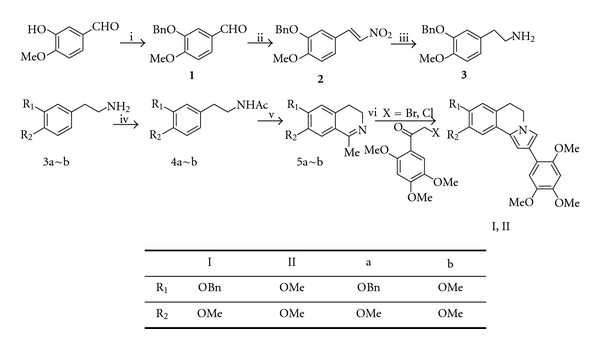
Reagents and conditions: (i) BnCl, K_2_CO_3_, EtOH, reflux, 5 h, 94%; (ii) CH_3_NO_2_, NH_4_OAc, AcOH, reflux, 4 h, 80.5%; (iii) LiAlH_4_, THF, reflux, 6 h, 84.9%; (iv) CH_3_COCl, Et_3_N, CH_2_Cl_2_, 0°C, 2 h, 4a: 81.9%, 4b: 90.7%; (v) POCl_3_, CH_2_Cl_2_, reflux, 3 h, 5a: 80.0%, 5b: 83.6%; (vi) 2-halogen-1-(2,4,5-trimethoxyphenyl)ethanone, CH_3_CN, K_2_CO_3_, reflux, 20 h, I: 74.7%, II: 72.6%.

Increasingly elegant synthetic routes have been developed. An efficient synthetic route for two compounds, 8-benzyloxy-9-methoxy-2-(2,4,5-trimethoxyphenyl)-5,6-dihydropyrrolo[2,1-a]isoquinoline (**I**) and 8,9-dimethoxy-2-(2,4,5-trimethoxyphenyl)-5,6-dihydro[2,1-a]isoquinoline (**II**), is mainly introduced in this study. The synthetic procedure is very valuable because it employs 5,6-dihydropyrrolo[2,1-a]isoquinolines as starting materials and represents an easy and direct approach to a wide variety of 3,4-dihydroisoquinolines. The synthetic strategy is outlined in Scheme 1.

Isovanillin was protected with benzyl chloride to get 3-benzyloxy-4-methoxy-benzaldehyde (1) with 84.4% yield [10], which was condensed with nitromethane giving 2-benzyloxy-1-methoxy-4-(2-nitrovinyl)-benzene (2) with 80.5% yield [11]. Compound **2** was then reduced with LiAlH_4_ to get 2-(3-benzyloxy-4-methoxyphenyl)-vinylamine (3) with 84.9% yield [12]. Treatment of **3a~b** with acetylchloride (n(**3a~b**) : n(CH_3_COCl) : n(Et_3_N) = 1 : 1.8 : 4.0) afforded acetamide (**4a~b**) with 81.9% and 90.7% yield, respectively, followed by cyclization with phosphorous oxychloride to get 3,4-dihydroisoquinoline **(5a~b)** with 80.0% and 83.6% yield (n(**4a**) : n(POCl_3_) = 1 : 8). A solution of **5a~b**, 2-bromo-1-(2,4,5-trimethoxy-phenyl)-ethanone and anhydrous K_2_CO_3_ in anhydrous acetonitrile was refluxed for 15 h. After a series of treatment, 5,6-dihydropyrrolo[2,1-a]isoquinoline **I** and **II** were obtained with 28.3% and 61.6% total yield, respectively.

## 2. Material and Methods

### 2.1. Analysis Means of Compounds

Melting points (uncorrected) were determined by a Gongyi X-4 apparatus. Infrared spectra(IR) were determined by Nicolet 550 spectrometer. NMR spectra were recorded by Bruker DRX500 or Bruker DRX400 spectrometer. All data were calibrated at *δ* 0.00 ppm for ^1^H spectra and ^13^C spectra from the original spectra (TMS). Low resolution mass spectra (LRMS) were recorded with an HP 6890/5973 GC-MS mass spectrometer. High resolution mass (HRMS) for unreported compounds were recorded with a Micromass GTC Gas Chromatography/TOF Mass spectrometer. All solvent were redistilled prior to use, unless otherwise stated, all other commercially available chemicals were used without further purification.

### 2.2. Chemical Synthesis

#### 2.2.1. 3-(benzyloxy)-4-methoxybenzaldehyde (1)

A mixture of isovanillin (10.0 g, 66 mmol), benzyl chloride (16 mL, 139 mmol), and anhydrous K_2_CO_3_ (6.5 g, 47 mmol) in EtOH (150 mL) was refluxed for 5 h. After being stirred, the reaction mixture was concentrated to dry and redissolved in 70 mL CH_2_Cl_2_, and then 5% aqueous NaOH (3 × 100 mL) was added. The organic layer was washed with brine (2 × 50 mL) and H_2_O (2 × 50 mL), dried with anhydrous Na_2_SO_4_, and evaporated to dryness. Needles were obtained after crystallization from MeOH/CH_2_Cl_2_ corresponding to 3-(benzyloxy)-4-methoxybenzaldehyde (15.0 g, 94%): m.p. 61~62°C (lit.^13^ m.p. 61~62°C); ^1^H NMR (400 MHz, CDCl_3_) *δ* 9.82 (s, 1H), 7.45~7.47 (m, 4H), 7.38 (t, 2H, *J* = 7.34 Hz), 7.32 (t, 1H, *J* = 7.34 Hz), 6.99 (d, 1H, *J* = 8.24), 5.19 (s, 2H), 3.96 (s, 3H); MS (EI, 70 ev) m/z: 242(M^+^), 92, 91, 79, 77, 65, 63, 51.

#### 2.2.2. (E)-2-(benzyloxy)-1-methoxy-4-(2-nitrovinyl)benzene (2)

A solution of compound **1** (10.0 g, 41 mmol), nitromethane (7 mL, 129 mmol) and NH_4_OAc (8.0 g, 104 mmol) in AcOH (125 mL) was refluxed for 4 h. After cooling, the mixture was diluted with H_2_O (100 mL) and extracted with CH_2_Cl_2_ (3 × 100 mL). The organic solution was washed with brine (2 × 100 mL) and H_2_O (2 × 100 mL), dried with anhydrous Na_2_SO_4_, and evaporated to dryness. Yellow needles were obtained from EtOH corresponding to (E)-2-(benzyloxy)-1-methoxy-4-(2-nitrovinyl)benzene (2) (9.6 g, 80.5%): m.p. 127~128°C (lit.^14^ m.p. 125~126°C)*；* ^1^H NMR (400 MHz, CDCl_3_) *δ* 7.91 (d, *J* = 13.6 Hz, 1H), 7.33~7.46 (m, 6H) 7.18 (dd, *J* = 2.0, 8.36 Hz, 1H), 7.03 (d, *J* = 2.0 Hz, 1H), 6.93 (d, *J* = 8.36 Hz, 1H), 5.17 (s, 2H), 3.95 (s, 3H); MS (EI, 70 ev) m/z: 285 (M^+^), 92, 91, 77, 65, 63, 51.

#### 2.2.3. 2-(3-(benzyloxy)-4-methoxyphenyl)ethanamine (3)

A solution of compound **2** (4.0 g, 14.0 mmol) in 14 mL of anhydrous THF was added dropwise to a well-stirred suspension of LiAlH_4_ (2.0 g, 52.8 mmol) in 50 mL of anhydrous THF and was refluxed for 6 h. After the solution was cooled, the excess reagent was destroyed by dropwise addition of EtOAc and 15% aqueous NaOH. After partial evaporation of the filtered portion, the aqueous solution was extracted with CH_2_Cl_2_ (3 × 30 mL), and the organic solution was washed with brine (2 × 20 mL) and H_2_O (2 × 20 mL), dried with anhydrous Na_2_SO_4_, and evaporated to dryness, and then 2-(3-(benzyloxy)-4-methoxyphenyl)ethanamine (3) (3.0 g, 84.9%) was obtained as an oil. ^1^H NMR (400 MHz, CDCl_3_) *δ* 6.73~7.44 (m, 8H), 5.12 (s, 2H), 3.85 (s, 3H), 2.85 (t, *J* = 6.7 Hz, 2H), 2.62 (t, *J* = 6.7 Hz, 2H), 2.20 (br s, 2H); MS (EI, 70 ev) m/z: 257 (M^+^), 229, 228, 167, 137, 92, 91, 65.

#### 2.2.4. N-(3-(benzyloxy)-4-methoxyphenethy)acetamide (4a)

A solution of 0.4 mL of acetyl chloride (5.6 mmol) in 5 mL anhydrous CH_2_Cl_2_ was added dropwise at 0°C to a solution of compound **3** (1.0 g, 3.88 mmol) and Et_3_N (1.7 mL, 12.26 mmol) in 20 mL anhydrous CH_2_Cl_2_, with stirring at 0°C for 2 h. After the mixture was stirred, 2.5% aqueous HCl was added and the organic solution was washed with brine (2 × 10 mL) and H_2_O (2 × 10 mL), dried with anhydrous Na_2_SO_4_, evaporated to dryness, and pale-yellow solid was obtained. Crude product was crystallized with EtOAc to afford *N*-(3-benzyloxy-4-methoxyphenylethyl)acetamide (0.94 g, 81.9%) as white crystals. m.p. 106~108°C (lit.^15^ m.p. 122~123°C); ^1^H NMR (400 MHz, CDCl_3_) *δ* 7.44 (d, *J* = 7.2 Hz, 2H), 7.36 (t, *J* = 7.2 Hz, 2H), 7.30 (d, *J* = 7.2 Hz, 1H), 6.84 (d, *J* = 8.8 Hz, 1H), 6.74 (d, *J* = 6.8 Hz, 2H), 5.14 (s, 2 H), 3.87 (s, 3H), 3.43 (q, *J* = 6.8, 12.8 Hz, 2H), 2.70 (t, *J* = 6.8 Hz, 2H), 1.88 (s, 3H).

#### 2.2.5. 6-(benzyloxy)-7-methoxy-1-methyl-3,4-dihydroiso-quinoline (5a)

A solution of 0.9 mL of POCl_3_ (9.8 mmol) in 6 mL anhydrous CH_2_Cl_2_ was added dropwise at 40°C to a solution of compound **4a** (0.4 g, 1.06 mmol) in 10 mL anhydrous CH_2_Cl_2_, with stirring at 40°C for 3 h, then was poured into ice-water mixture, 2.5% aqueous NaOH was added to make pH about 12, the aqueous solution was extracted with CH_2_Cl_2_ (3 × 20 mL), and the organic solution was washed with brine (2 × 10 mL) and H_2_O (2 × 10 mL), dried with anhydrous Na_2_SO_4_, evaporated to dryness and solid was obtained. The crude product was purified with a silica gel column (Petroleum : EtOAc(v/v) = 3 : 1, 200~300 H) to afford 6-(benzyloxy)-7-methoxy-1-methyl-3,4-dihydroisoquinoline (5a) (0.24 g, 80%) as brick red crystals. m.p. 95~96°C; ^1^H NMR (500 MHz, CDCl_3_) *δ* 7.30~7.44 (m, 5H), 7.01 (s, 1H), 6.7 (s, 1H), 5.17 (s, 2H), 3.90 (s, 3H), 3.61 (t, *J* = 7.2 Hz, 2H), 2.57 (m, *J* = 1.3, 7.5 Hz, 2H), 2.35 (t, *J* = 1.3 Hz, 3H).

#### 2.2.6. 8-benzyloxy-9-methoxy-2-(2,4,5-trimethoxyphenyl)-5,6-dihydropyrrolo[2,1-a]isoquinoline (I)

To a solution of 0.52 g compound 5a (1.85 mmol) in 15 mL anhydrous CH_3_CN was added 0.45 g 2-bromo-1-(2,4,5-trimethoxy-phenyl)-ethanone (1.85 mmol). The reaction mixture was stirred at 85°C for 10 h, then 0.38 g anhydrous K_2_CO_3_ (2.75 mmol) was added and continued to stir for another 10 h. After that the mixture was poured into 15 mL brine and extracted with CH_2_Cl_2_ (3 × 15 mL), the combined organic layers were dried with anhydrous Na_2_SO_4_, evaporated to dry, and brown oil was obtained. The crude product was purified with a silicagelcolumn (Petroleum : EtOAc(v/v) = 2 : 1, 200~300 H) to afford 8-benzyl-9-methoxy-2-(2,4,5-trimethoxyphenyl)-5,6-dihydropyrrolo[2,1-a]isoquinoline (I) (0.65 g, 74.7%) as offwhite sheet solid. m.p. 128°C; IR (KBr) **ν**: 2993, 2934, 2830, 1614, 1568, 1529, 1508, 1453,1427, 1365, 1336, 1274, 1166, 1130, 1035, 848, 810, 784, 738, 695 cm^−1^; ^1^H NMR (500MHz, CDCl_3_) *δ* 7.29–7.46 (m, 5H), 7.12 (d, *J* = 1.6 Hz, 1H), 7.11 (s, 1H), 7.10 (s, 1H), 6.73 (s, 1H), 6.69 (d, *J* = 1.6 Hz, 1H), 6.60 (s, 1H), 5.14 (s, 2H), 4.05 (t, *J* = 6.6 Hz, 2H), 3.94 (s, 3H), 3.91 (s, 3H), 3.91 (s, 3H), 3.88 (s, 3H), 2.95 (t, *J* = 6.6 Hz, 2H); ^13^C NMR (500 MHz, CDCl_3_) *δ*: 29.61, 44.91, 56.96, 56.97, 57.14, 57.43, 72.14, 99.34, 102.13, 107.32, 112.83, 115.18, 117.55, 120.83, 121.02, 123.60, 123.78, 128.03, 128.03, 128.50, 129.21, 129.21, 130.31, 137.98, 144.06, 147.25, 148.13, 149.82, 151.07; MS (LC-MS) m/z: 472 (M+1)^+^, 367, 318, 273; HRMS (ESI-Q-TOF) calcd for C_29_H_29_NO_5_ [M+1]^+^ 472.4856, found 472.4819.

#### 2.2.7. N-(3,4-dimethoxyphenethyl)acetamide (4b)

A solution of 7.6 mL of acetyl chloride (0.11 mol) in 10 mL anhydrous CH_2_Cl_2_ was added dropwise at 0°C to a solution of compound **3b** (10 mL, 0.059 mol) and Et_3_N (32.8 mL, 0.23 mol) in 25 mL anhydrous CH_2_Cl_2_, with stirring at 0°C for 2 h. After the mixture was stirred, 2.5% aqueous HCl was added and the organic solution was washed with brine (2 × 30 mL) and H_2_O (2 × 20 mL), dried with anhydrous Na_2_SO_4_, evaporated to dryness, and yellow solid was obtained. Crude product was crystallized with EtOAc to afford N-(3,4-dimethoxyphenethyl) acetamide (4b) (11.8 g, 90.7%) as yellow crystals. m.p. 85~86°C (lit.^16^ m.p. 94°C); IR (KBr) **ν**: 1642.54 cm^−1^ (–C=O), 3301.49 cm^−1^ (–NH–).

#### 2.2.8. 6,7-dimethoxy-1-methyl-3,4-dihydroisoquinoline (5b)

A solution of 9.8 mL of POCl_3_ (0.1 mol) in 40 mL anhydrous CH_2_Cl_2_ was added dropwise at 40°C to a solution of compound **4b** (3.0 g, 13.4 mmol) in 30 mL anhydrous CH_2_Cl_2_, with stirring at 40°C for 3 h, then was poured into ice-water mixture; 2.5% aqueous NaOH was added to make pH about 12, the aqueous solution was extracted with CH_2_Cl_2_ (3 × 60 mL), and the organic solution was washed with brine (2 × 50 mL) and H_2_O (2 × 50 mL), dried with anhydrous Na_2_SO_4_, evaporated to dryness, and solid was obtained. The crude product was purified with a silica gel column (Petroleum : EtOAc(v/v) = 1 : 1, 200~300 H) to afford 6,7-dimethoxy-1-methyl-3,4-dihydroisoquinoline (5b) (2.3 g, 83.6%) as brick red crystals. m.p. 98~99°C (lit.^17^ m.p. 85~96°C)*；* ^1^H NMR (500 MHz, CDCl_3_) *δ* 6.99 (s, 1H), 6.89 (s, 1H), 3.92 (s, 3H), 3.91 (s, 3H), 3.63 (m, *J* = 1.4, 7.5 Hz, 2H), 2.63 (t, *J* = 7.5 Hz, 2H), 2.36 (t, *J* = 1.4 Hz, 3H).

#### 2.2.9. 8,9-dimethoxy-2-(2,4,5-trimethoxyphenyl)-5,6-dihydro[2, 1-a]isoquinoline (II)

To a solution of 1.5 g compound 5b (7.32 mmol) in 20 mL anhydrous CH_3_CN was added 1.78 g 2-bromo-1-(2,4,5-trimethoxy-phenyl)-ethanone (7.34 mmol). The reaction mixture was stirred at 85°C for 10 h, then 1.52 g anhydrous K_2_CO_3_ (11.0 mmol) was added and continued to stir for another 10 h. After that the mixture was poured into 30 mL brine and extracted with CH_2_Cl_2_ (3 × 30 mL), the combined organic layers were dried with anhydrous Na_2_SO_4_, evaporated to dryness, and brown oil was obtained. The crude product was purified with a silica gel column (Petroleum : EtOAc(v/v) = 2 : 1, 200~300 H) to afford 8,9-dimethoxy-2-(2,4,5-trimethoxyphenyl)-5,6-dihydro[2,1-a]isoquinoline (II) (0.65 g, 72.6%) as gray solid. m.p. 137~138°C; IR (KBr) **ν**: 2993, 2934, 2836, 1608, 1560, 1530, 1508, 1484, 1397, 1272, 1212, 1126, 1036, 808, 776 cm^−1^; ^1^H NMR (500 MHz, CDCl_3_) *δ*: 3.01 (t, *J* = 6.6 Hz, 2H), 3.87 (s, 3H), 3.88 (s, 3H), 3.91 (s, 3H), 3.92 (s, 3H), 3.95 (s, 3H), 4.07 (t, *J* = 6.6 Hz, 2H), 6.60 (s, 1H), 6.69 (d, *J* = 1.7 Hz, 1H), 6.70 (s, 1H), 7.08 (s, 1H), 7.12 (s, 1H), 7.13 (d, *J* = 1.7 Hz, 1H); ^13^C NMR (500 MHz, CDCl_3_) *δ*: 29.07, 44.28, 56.08, 56.15, 56.30, 56.49, 56.78, 98.68, 106.08, 101.36, 111.51, 112.19, 116.90, 120.12, 120.36, 122.43, 122.93, 129.70, 143.40, 147.38, 147.47, 148.38, 150.42; DEPT 135 (500 MHz, CDCl_3_) *δ*: two –CH_2_ (29.06, 44.28), five –CH_3_ (56.07, 56.14, 56.29,56.48,56.76), six –CH (98.62, 101.34, 106.05, 111.47, 112.15, 120.11); MS (LC-MS) m/z: 396 (M+1)^+^, 371, 276; HRMS (ESI-Q-TOF) calcd for C_23_H_25_NO_5_ [M+1]^+^ 396.4852, found 396.4884.

## 3. Results

**Figure 1:**
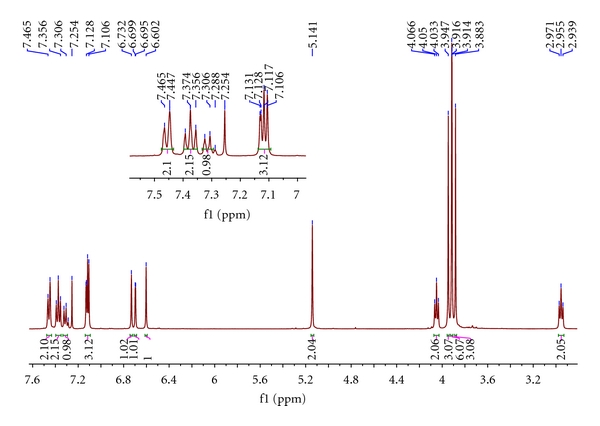
^1^H NMR spectrum of the **I**. Inserted figure is the magnification of the part of 7.00–7.50 of chemical shift.

**Figure 2:**
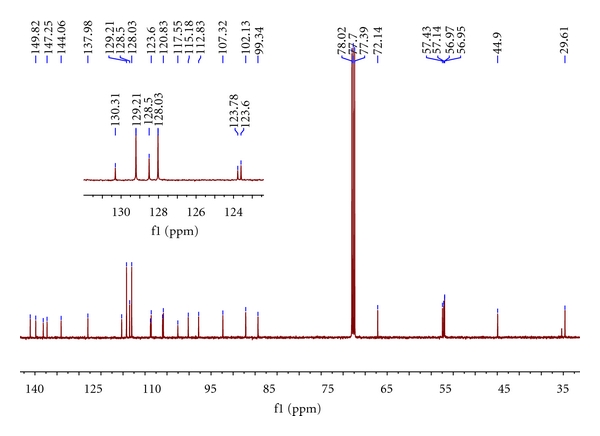
^13^C NMR spectrum of the **I**. Inserted figure is the magnification of the part of 120.00–135.00 of chemical shift.

**Figure 3:**
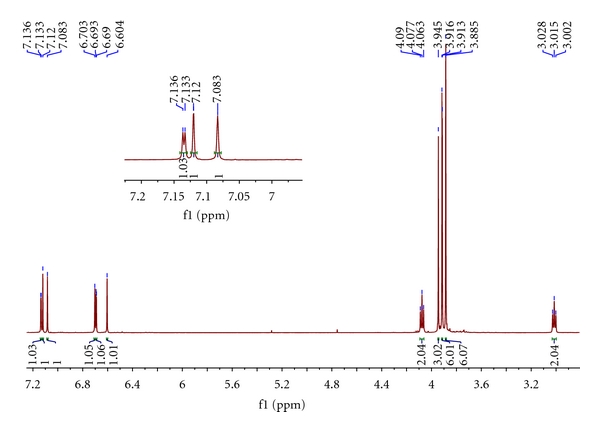
^1^H NMR spectrum of the **II**. Inserted figure is the magnification of the part of 7.00–7.20 of chemical shift.

**Figure 4:**
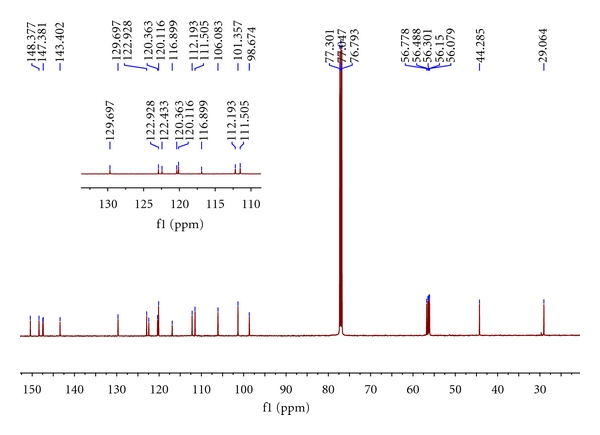
^13^C NMR spectrum of the **II**. Inserted figure is the magnification of the part of 110.00–130.00 of chemical shift.

 The target compounds **I** and **II** had been synthesized by our route and their structures were determined by interpretation of spectral data. The ^1^H NMR and ^13^C NMR spectra of them were assigned as indicated in Figures 1, 2, 3, and 4.

An initial ^1^H-NMR spectrum of **I** (in CDCl_3_) revealed four –OMe–H signals at 3.94 (s, 3H), 3.91 (s, 3H), 3.91 (s, 3H), 3.88 (s, 3H). These peaks are the featured signals of the –OMe–. 2.95 and 4.05 *doublets* (*J* = 6.6 Hz) indicate –CH_2_N– and –CH_2_– moieties connected with it in the isoquinoline ring. It can be seen that the distinguishing feature of Ar–CH_2_O 5.14 (s, 2H) is shown in Figure 1. There are several groups of signals in the aromatic region; they are 7.12 (d, *J* = 1.6 Hz, 1H), 7.11 (s, 1H), 7.10 (s, 1H), 6.73 (s, 1H), 6.69 (d, *J* = 1.6 Hz, 1H), and 6.60 (s, 1H), respectively. Among them, 7.12 (d, *J* = 1.6 Hz, 1H) and 6.69 (d, *J* = 1.6 Hz, 1H) are the signals in the pyrrole ring; this can be estimated from the peak type. Since Ar–H in the Ar–CH_2_O are influenced by other protons more slightly, they will overlap together and show the multiplet in the spectra. So 7.29–7.46 (m, 5H) is the signal of Ar–H in the Ar–CH_2_O. A molecular formula of C_29_H_29_NO_5_, resulted from HR-MS data of **I.** The ^13^C NMR spectrum of **I** displayed twenty-seven signals, which represented all twenty-nine C-atoms, eighteen of which were assignable to three aromatic-C moieties and accounted for sixteen spectral signals. Of the remaining eleven signals, four were from OMe (56.96, 56.97, 57.14, 57.43 ppm), and seven were from isoquinoline and pyrrole ring C-atoms.

 NMR data of **II** (see Figures 3 and 4) indicated a C_23_H_25_ framework, which HR-MS analysis expanded to a molecular formula of C_23_H_25_NO_5_. The simplest assumed relationship between the two isoquinoline, **I** as an BnO-substituted **II**, was reinforced by characterization of the NMR data, which exhibited many similar signals. Specifically, too many shifts of H and C resonances are very similar to each other which proved the basic framework between **I** and **II**. The NMR signals which distinguished **I** from **II** were those of three aromatic protons appropriate for Ar–H (7.29–7.46 ppm, m, 5H) and –CH_2_– in the Ar–CH_2_O. The remaining distinguishing feature was the number of –OMe– signal in ^13^C NMR at 56-57 ppm.

## 4. Discussions

**I** and **II** from 1-methyl-3,4-dihydroisoquinoline and 2,4,5-trimethoxy-*α*-halogen-acetophenone were obtained with high yields under mild conditions for the first time. This novel method, as the key reaction step, provides a general and highly efficient method for the preparation of 5,6-dihydropyrrolo[2,1-a]isoquinolines. We envisaged that the 5,6-dihydropyrrolo[2,1-a]isoquinolines could be constructed by the formation of quaternary ammonium salt, and subsequent lactonization in the presence of anhydrous K_2_CO_3_. The negative carbon ion of 1-methyl-3,4-dihydroisoquinoline is also active in the Knorr reaction. Both 2,4,5-trimethoxy-*α*-bromoacetophenone and 2,4,5-trimethoxy-*α*-chloracetophenone were employed. We found that the yield of the former is about 5% higher than the later. Therefore, 2,4,5-trimethoxy-*α*-bromoacetophenone is used in the synthesis of **I** and **II**.

## 5. Conclusion

Based on the facile synthetic route depicted in Scheme 1, two novel scaffolds for synthesis of lamellarin analogues 8-benzyloxy-9-methoxy-2-(2,4,5-trimethoxyphenyl)-5,6-dihydropyrrolo[2,1-a] isoquinoline (**I**) and 8,9-dimethoxy-2-(2,4,5-trimethoxyphenyl)-5,6-dihydro[2,1-a]isoquinoline (**II**) were obtained under mild condition. These two compounds are characterized by ^1^H NMR, ^13^C NMR, IR spectrum, and melting points. The products are stable and may be expected to exhibit biological activities to some extend.
